# Is There a Role for Hepatobiliary Scintigraphy in Thermal Ablation of Hepatocellular Carcinoma?

**DOI:** 10.3390/cancers18020322

**Published:** 2026-01-20

**Authors:** Niek Wijnen, Joep de Bruijne, Rutger C. G. Bruijnen, Emma Ruijs, Hugo W. A. M. de Jong, Marnix G. E. H. Lam, Maarten L. J. Smits

**Affiliations:** 1 Department of Radiology and Nuclear Medicine, University Medical Center Utrecht, 3584 CX Utrecht, The Netherlands; 2 Department of Gastroenterology and Hepatology, University Medical Center Utrecht, 3584 CX Utrecht, The Netherlands

**Keywords:** hepatocellular carcinoma, hepatobiliary scintigraphy, liver cirrhosis, liver function, thermal ablation, segmentation

## Abstract

**Purpose**: Hepatobiliary scintigraphy (HBS) provides quantitative assessment of (future remnant) liver function, aiding clinical decision-making for surgical resection and radioembolization of hepatocellular carcinoma (HCC). However, its role for thermal ablation remains unexplored. This pilot study aimed to explore the potential role of HBS in guiding patient selection and risk stratification for thermal ablation. **Methods**: All HCC patients who underwent thermal ablation between January 2021 and August 2025 and had HBS performed prior to ablation were retrospectively reviewed. Ablated non-tumor liver volumes (i.e., volume of ablated healthy parenchyma) were quantified using 3D segmentation. Absolute ablated liver function (i.e., the proportion of total HBS-derived liver function ablated) was also assessed. Clinical outcomes included changes in clinical scores (e.g., Child–Pugh) and the occurrence of hepatic decompensation after ablation. **Results**: Nine patients (13 tumors) were included. Median global HBS-derived liver function was 3.2%/min/m^2^ (range 1.6–6.8%/min/m^2^). Three patients developed hepatic decompensation > 3 months after ablation, unlikely related to thermal ablation itself. The patient with the lowest baseline function (1.6%/min/m^2^) tolerated ablation without hepatic decompensation. Median ablated non-tumor liver volume was 14.4 cm^3^ (range 3.1–46.7 cm^3^), corresponding to a median of 0.9% (range 0.2–3.6%) of total liver volume ablated per lesion. Median absolute ablated function was 0.05%/min/m^2^ (range 0.02–0.21%/min/m^2^). **Conclusions**: Thermal ablation was feasible and well tolerated even in patients with severely impaired liver function. Routine pre-ablation HBS does not appear necessary for thermal ablation of HCC, as only a small percentage of total functional liver volume is ablated.

## Simple Summary

Thermal ablation is an established minimally invasive treatment for hepatocellular carcinoma, but it inevitably affects surrounding healthy liver tissue, which may be clinically relevant in patients with limited hepatic reserve. Hepatobiliary scintigraphy is an imaging technique that enables quantitative assessment of global and regional liver function, yet its role in the setting of thermal ablation has not been previously explored. In this pilot study, we evaluated patients who underwent thermal ablation for hepatocellular carcinoma and had hepatobiliary scintigraphy performed prior to treatment. We assessed procedural safety and estimated the amount of functional liver tissue affected by ablation. Our results show that thermal ablation resulted in only minimal loss of functional liver parenchyma (median 0.9% of total liver volume per ablated lesion) and was feasible even in patients with severely impaired liver function. These findings suggest that routine use of hepatobiliary scintigraphy prior to thermal ablation is not required.

## 1. Introduction

Hepatobiliary scintigraphy (HBS) with technetium-99 m (^99m^Tc)-mebrofenin enables quantitative assessment of hepatic and biliary function [1,2]. It is increasingly employed to guide patient selection for liver-directed therapies, particularly for surgical resection and transarterial radioembolization (TARE) [3,4]. By providing both regional and global assessments of liver function, HBS improves risk stratification and supports individualized treatment planning [5,6]. HBS can identify patients at risk of posthepatectomy liver failure by quantifying the functional reserve of the future liver remnant (FLR), thereby guiding the need and timing for FLR hypertrophy-inducing procedures [7,8]. In TARE, HBS is used to estimate the functional reserve of the non-treated liver, thereby aiding in patient selection, dose planning, and prediction of radioembolization-induced liver disease [3,9].

Despite its emerging role in clinical decision-making for liver-directed therapies, the role of HBS remains unexplored in the context of thermal ablation. Thermal ablation is a first-line therapy for hepatocellular carcinoma (HCC) up to 3 cm and is increasingly used for larger lesions (>3 cm) [10,11,12,13]. With ongoing advances in image-guided ablation techniques, thermal ablation is increasingly applied in more complex cases. Although generally considered safe, a retrospective study of 498 HCC patients undergoing microwave ablation (MWA) reported post-procedural liver dysfunction (defined as a ≥2-point increase in Child–Pugh score within three days) in 14% of patients, with higher rates in those with large ablation volumes (≥22.5 cm^3^) and baseline Child–Pugh B status [14]. These findings suggest that pre-ablation functional assessment with HBS may provide added value in these higher-risk groups. However, the impact of thermal ablation on HBS-derived liver function remains unexplored, and outcomes in patients with severely compromised hepatic reserve (<2.7%/min/m^2^ on HBS) have not been described before.

Therefore, the aim of this pilot study was to explore whether HBS has a potential role in thermal ablation by evaluating clinical outcomes in patients who underwent thermal ablation for HCC and had prior HBS (performed for another indication). We assessed the safety of thermal ablation in patients with impaired liver function and correlated outcomes with volumetric measurements of functionally ablated liver tissue.

## 2. Methods

### 2.1. Ethical Approval

Data for the present analysis were obtained from the Minimally Invasive Thermal Ablation (MISTRAL) registry, an ongoing prospective database that includes patients undergoing thermal ablation of liver tumors at the University Medical Center Utrecht (Utrecht, The Netherlands). The registry was approved by the local institutional review board (approval no. 21/709), and all patients provided written informed consent for the use of their data for research purposes.

### 2.2. Patients

All consecutive patients who underwent percutaneous thermal ablation for HCC between January 2021 and August 2025, and who had HBS imaging for another indication, with no substantial change in liver function between HBS and ablation (as indicated by stable laboratory values or an unchanged Child–Pugh classification), were retrospectively reviewed. Patients were excluded if: (1) thermal ablation was combined with another local treatment; (2) another local treatment was performed between the HBS imaging and thermal ablation; (3) cholestasis, as determined by the treating hepatologist based on clinical symptoms and abnormal liver laboratory findings (including elevated serum bilirubin and alkaline phosphatase levels), was observed at the time of HBS; or (4) follow-up data were unavailable.

### 2.3. Hepatobiliary Scintigraphy

Immediately after intravenous administration of ^99m^Tc-mebrofenin (Bromo-Biliaron, GE Healthcare, Chicago, IL, USA), dynamic anterior and posterior images were acquired using a Symbia T16 SPECT/CT system (Siemens, Berlin, Germany) (36 frames, 10 s/frame; 128 × 128 matrix; energy window: 140 keV ± 7.5%) [1]. Subsequently, a SPECT/CT acquisition was performed (60 projections, 8 s/projection; 128 × 128 matrix; energy window: 140 keV ± 7.5%) to evaluate regional hepatic function. Finally, a second dynamic phase was obtained (15 frames, 60 s/frame; 128 × 128 matrix; energy window: 140 keV ± 7.5%) to assess biliary excretion, followed by a low-dose CT scan for anatomical reference and attenuation correction.

### 2.4. Thermal Ablation

Percutaneous thermal ablation procedures were performed using the Hepatic Arteriography and C-arm CT-Guided Ablation (HepACAGA) technique, as previously described [15]. The HepACAGA technique involves intra-arterial administration of contrast into the hepatic artery during C-arm CT acquisition (i.e., C-arm CT hepatic arteriography) to enhance intraprocedural visualization of the tumor and ablation zone [16]. All ablations were performed using MWA with the Emprint^®^ HP system (Medtronic, Dublin, Ireland).

### 2.5. Liver Function and Volumes

**Figure 1:**
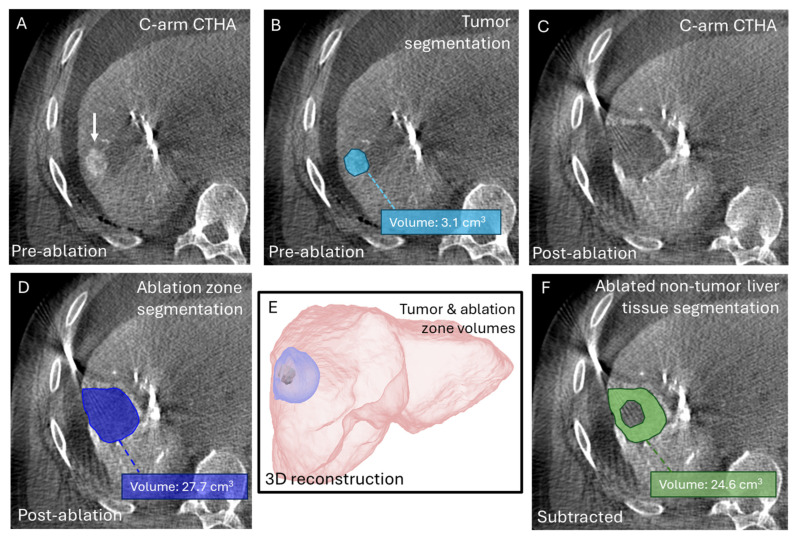
Schematic illustration of the segmentation workflow. (**A**) Pre-ablation C-arm CT hepatic arteriography (CTHA) image showing the target HCC (18 mm) (white arrow); (**B**) Tumor segmentation (cyan) performed on pre-ablation C-arm CTHA; (**C**) Post-ablation C-arm CTHA after thermal ablation, showing the hypodense ablation zone surrounded by a hyperemic rim; (**D**) Ablation zone segmentation (blue) performed on post-ablation C-arm CTHA; (**E**) 3D reconstruction of segmented tumor (cyan) and ablation zone (blue) volumes within the liver (red); (**F**) Subtraction of the tumor and ablation zone volumes results in the volume of ablated non-tumor liver tissue (green).

Total and regional liver function were derived from HBS and expressed as [%/min/m^2^]. Total liver volume was also obtained from HBS. Impaired liver function was defined as HBS values < 2.7%/min/m^2^ [8]. Tumor and ablation zone volumes were quantified using semi-automatic segmentation on intraprocedural pre-ablation and post-ablation C-arm CT hepatic arteriography scans, respectively (Figure 1). All segmentations were performed using the built-in segmentation tools of the PACS (Picture Archiving and Communication System IDS7 v27.1) software (Sectra, Linköping, Sweden).

To estimate the amount of healthy liver parenchyma affected by treatment, the volume of ablated non-tumor (i.e., healthy liver parenchyma) liver tissue ($V_{\mathrm{ablated}\;non\text{-}tumor}$) was calculated by subtracting the tumor volume(s) ($V_{\mathrm{tumor}}$) from the ablation zone volume(s) ($V_{\mathrm{ablation}\;\mathrm{zone}}$) (Figure 1F).

(1) $$V_{\mathrm{ablated}\;non\text{-}tumor}=\sum_{i}\left(V_{\mathrm{ablation}\;\mathrm{zone},i}-V_{\mathrm{tumor},i}\right)$$

The percentage of ablated liver volume ($\%V_{\mathrm{ablated}\;\mathrm{liver}}$) was obtained by dividing the total ablation zone volume (in case of multiple ablations, the ablation zone volumes were summed) by the total liver volume.

(2) $$\%V_{\mathrm{ablated}\;\mathrm{liver}}=\frac{\sum_{i}V_{\mathrm{ablation}\;\mathrm{zone},i}}{V_{\mathrm{total}\;\mathrm{liver}}}\times 100$$

The percentage of ablated non-tumor liver volume ($\%V_{\mathrm{ablated}\;non\text{-}tumor}$) was determined by dividing the volume of ablated non-tumor liver tissue by the total liver volume.

(3) $$\%V_{\mathrm{ablated}\;non\text{-}tumor}=\frac{V_{\mathrm{ablated}\;non\text{-}tumor}}{V_{\mathrm{total}\;\mathrm{liver}}}\times 100$$

Finally, the absolute ablated liver function (%/min/m^2^)—representing the estimated proportion of total liver function lost due to ablation—was calculated by multiplying the total HBS-derived liver function by the fraction of non-tumor liver volume ablated ($\%V_{\mathrm{ablated}\;non\text{-}tumor}$). This calculation assumed a homogeneous distribution of liver function across the hepatic parenchyma.

### 2.6. Clinical Parameters

Baseline clinical parameters assessed included albumin–bilirubin (ALBI) score and Model for End-Stage Liver Disease (MELD) score. During follow-up, patients were monitored for changes in Child–Pugh or Barcelona Clinic Liver Cancer (BCLC) staging, clinical signs of hepatic decompensation (e.g., ascites, hepatic encephalopathy), disease recurrence or progression on imaging, and overall survival.

Hepatic decompensation was considered probably related to thermal ablation when the following criteria were met: (1) occurrence within 3 months post-ablation, and (2) absence of multifocal disease progression on imaging.

### 2.7. Statistics

Categorical variables are presented as frequencies and percentages, while continuous variables are expressed as medians with range. Scatterplots are presented with Pearson correlation coefficients (*r*). Due to the limited sample size, additional formal comparative statistical analyses were not performed.

## 3. Results

### 3.1. Patients

**Figure 2:**
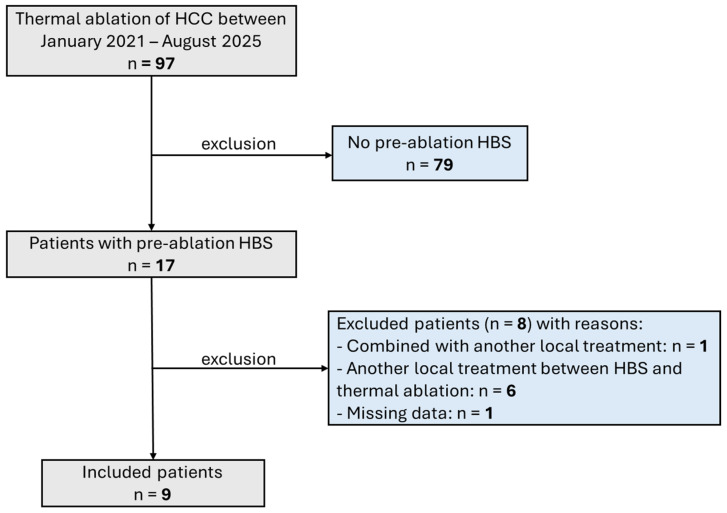
Flowchart demonstrating patient selection. HBS = Hepatobiliary scintigraphy.

After applying exclusion criteria, nine patients (13 HCC tumors), who underwent thermal ablation and had pre-ablation HBS (performed to assess candidacy for surgery or TARE, or for post-treatment liver function evaluation), were included (Figure 2). Baseline characteristics are summarized in Table 1. Cirrhosis was present in all HCC patients, with metabolic dysfunction-associated steatohepatitis (MASH) being the most common cause (4/9, 44%), followed by post-alcoholic cirrhosis (2/9, 22%). Six of nine patients had Child–Pugh class B7 or higher, one patient had ECOG performance status 3, and five patients had a MELD score ≥ 10.

Median liver function derived from HBS was 3.2%/min/m^2^ (range 1.6–6.8%/min/m^2^). Impaired liver function (<2.7%/min/m^2^) was observed in 3/9 patients (33%). The median time interval between HBS and ablation was 1.0 months (range 0.2–16.3 months), with seven patients having an interval < 3 months. Median post-ablation follow-up was 7 months (range 3–21 months).

### 3.2. Volumetric and Liver Function Analysis

Median total liver volume ($V_{\mathrm{total}\;\mathrm{liver}}$) was 1450 cm^3^ (range 766–2009 cm^3^) (Table 2). Median tumor diameter was 22 mm (range 9–47 mm), and the median tumor volume ($V_{\mathrm{tumor}}$) was 5.6 cm^3^ (range 0.3–30.4 cm^3^). Median ablation volume ($V_{\mathrm{ablation}\;\mathrm{zone}}$) was 19.2 cm^3^ (range 11.7–77.1 cm^3^) per tumor and 34.0 cm^3^ (range 17.2–77.1 cm^3^) per patient. The corresponding median percentage of ablated liver volume ($\%V_{\mathrm{ablated}\;\mathrm{liver}}$) was 1.3% (range 0.7–5.9%) per tumor and 3.0% (range 1.1–5.9%) per patient.

Median ablated non-tumor volume ($V_{\mathrm{ablated}\;non\text{-}tumor}$) was 14.4 cm^3^ (range 3.1–46.7 cm^3^) per tumor and 28 cm^3^ (range 12.7–46.7 cm^3^) per patient. The corresponding median percentage of ablated non-tumor liver volume ($\%V_{\mathrm{ablated}\;non\text{-}tumor}$) was 0.9% (range 0.2–3.6%) per tumor and 2.2% (range 0.8–3.7%) per patient. Median absolute ablated function was 0.04%/min/m^2^ (range 0.01–0.21%/min/m^2^) per tumor and 0.05%/min/m^2^ (range 0.02–0.21%/min/m^2^) per patient.

**Figure 3:**
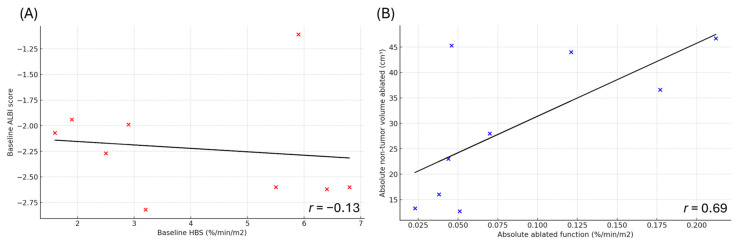
Scatterplots with linear trend lines. Each data point represents an individual patient. (**A**) Relationship between baseline hepatobiliary scintigraphy (HBS)–derived liver function (%/min/m^2^) and baseline albumin–bilirubin (ALBI) score, demonstrating poor correlation (*r* = −0.13). (**B**) Relationship between non-tumor liver volume ablated (cm^3^) and absolute ablated liver function (%/min/m^2^), demonstrating moderate correlation (*r* = 0.69).

Correlation analysis demonstrated a poor relationship between HBS-derived liver function and ALBI score (*r* = −0.13) (Figure 3A), whereas the correlation between absolute ablated liver function and ablated non-tumor liver volume was moderate (*r* = 0.69) (Figure 3B).

### 3.3. Follow-Up Analysis

A detailed follow-up analysis of patients who developed hepatic decompensation after ablation is provided below. None met the pre-defined criteria for ablation-related decompensation, indicating that decompensation was unlikely attributable to the thermal ablation procedure.

#### 3.3.1. Patients with Impaired Liver Function (<2.7%/min/m^2^)

Among the three patients with impaired baseline liver function, one developed hepatic decompensation (Patient #3, 2.5%/min/m^2^). One month after thermal ablation of a 24 mm lesion (non-tumor volume ablated 13.3 cm^3^, representing 0.9% of total liver volume), this patient developed ascites and tumor progression on imaging. At 4 months follow-up, imaging showed multifocal HCC progression (BCLC B→D). With worsening ECOG (1→2) and Child–Pugh scores (B7→B8), systemic therapy was not indicated. The patient died seven months post-ablation.

**Figure 4:**
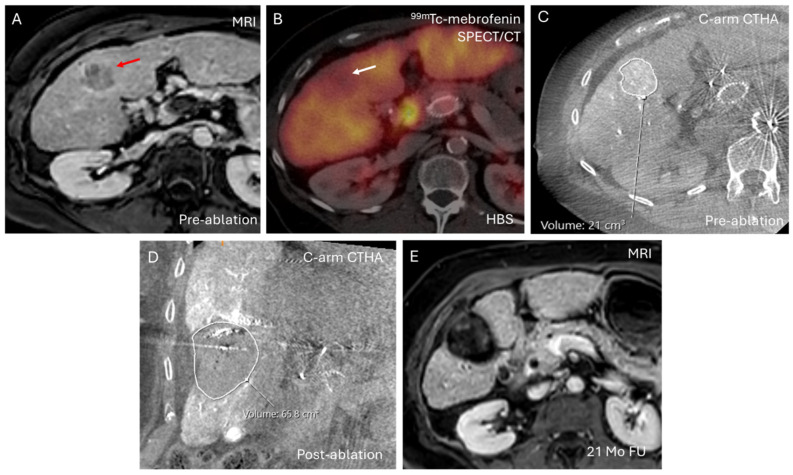
Case example of a patient with severely impaired liver function (1.6%/min/m^2^) who underwent ablation of a large HCC (maximum diameter: 40 mm). (**A**) Pre-treatment MRI (T1-weighted + gadolinium) showing the target lesion (red arrow); (**B**) ^99m^Tc-mebrofenin SPECT/CT from hepatobiliary scintigraphy (HBS) demonstrating a photopenic area (“cold spot”) corresponding to the tumor (white arrow); (**C**) Intraprocedural C-arm CT hepatic arteriography (C-arm CTHA) depicting the target HCC (volume = 21.0 cm^3^); (**D**) Post-ablation C-arm CTHA (coronal) showing the ablation zone (volume = 65.8 cm^3^); (**E**) Follow-up MRI (T1-weighted + gadolinium) at 21 months demonstrating no tumor recurrence or progression. Despite the severely impaired baseline liver function and the relatively large ablation volume, the patient showed no signs of post-ablation liver failure.

Patient #1 (1.6%/min/m^2^), who had the lowest baseline liver function, underwent thermal ablation of a 40 mm lesion (21.0 cm^3^) (see Figure 4). The ablation zone measured 65.8 cm^3^, with a non-tumor ablation volume of 44.8 cm^3^ (2.9% of total liver volume). The Child–Pugh score increased from B7 to B8 during follow-up due to elevated bilirubin; however, this patient remained clinically stable without hepatic decompensation and recurrence-free up to last follow-up visit 21 months after ablation.

Patient #2 (1.9%/min/m^2^) remained clinically stable and underwent liver transplantation four months after thermal ablation.

#### 3.3.2. Patients with Preserved Liver Function (≥2.7%/min/m^2^)

Among six patients with preserved liver function (median 5.7%/min/m^2^, range 2.9–6.8%/min/m^2^), two developed hepatic decompensation during follow-up.

Patient #5 (3.2%/min/m^2^) underwent ablation of a 12 mm lesion with a non-tumor ablation volume of 16.0 cm^3^ (1.2% of total liver volume). Six months post-ablation, this patient developed ascites (Child–Pugh B8→B9). No radiological HCC progression was observed. The patient died 12 months post-procedure.

Patient #6 (5.5%/min/m^2^) underwent ablation of three lesions (total tumor volume: 17.4 cm^3^) with a non-tumor volume ablated of 44.0 cm^3^ (2.2% of total liver volume). Multifocal HCC recurrence with severe ascites occurred within 4 months after ablation (Child–Pugh B7→B9). The patient died six months post-ablation.

The remaining four patients remained clinically stable during follow-up, with no clinical or radiological evidence of hepatic decompensation or tumor recurrence following ablation.

## 4. Discussion

This pilot study explored the association between pre-ablation HBS-derived liver function and post-ablation clinical outcomes in patients with HCC. The most important findings are: (1) in none of the patients was hepatic decompensation likely related to the thermal ablation procedure itself; (2) the proportion of non-tumor liver parenchyma ablated per lesion was very small (median 0.9%, range 0.2–3.6%); and (3) thermal ablation was feasible even in patients with severely impaired total liver function (lowest baseline value 1.6%/min/m^2^).

The small proportion of functional liver parenchyma ablated per tumor (median 0.9% of total liver volume) likely explains why locoregional thermal ablation remains feasible even in patients with severely impaired liver function. Notably, patient #1, who had the lowest baseline liver function (1.6%/min/m^2^), successfully underwent ablation of a 40 mm lesion without post-procedural hepatic decompensation, despite the ablation of 44.8 cm^3^ of non-tumor liver tissue (2.9% of total liver volume). In contrast with surgical resection, where segmentectomy can precipitate liver failure in patients with compromised hepatic reserve, ablation spares enough functional parenchyma to maintain postoperative liver function [17,18,19]. As such, the commonly used surgical cut-off of at least 2.7%/min/m^2^ FLR function may be overly conservative when applied to thermal ablation.

The relatively small ablation volumes observed in this study may be partly attributable to the use of the HepACAGA technique, which enables accurate intraprocedural tumor visualization and ablation zone margin confirmation [11,15]. This allows precise intraprocedural assessment of the ablation margins, ensuring a minimal margin of ≥5 mm (generally considered adequate to reduce the risk of post-ablation local tumor recurrence) [20,21,22,23,24,25]. Consequently, the need to create unnecessarily large ablation zones to compensate for uncertainty in margin assessment is avoided. In contrast, many centers still perform thermal ablation using conventional CT guidance based on anatomical landmarks, in which ablation zones may be intentionally enlarged to compensate for uncertainty in ablation margins [26,27,28]. In such workflows, a relatively greater proportion of non-tumor liver parenchyma may be ablated, potentially increasing the risk of post-procedural liver dysfunction, particularly in high-risk patients with limited functional reserve.

In radiation segmentectomy, the treated liver volume and associated functional impact may be substantially larger than with thermal ablation. A retrospective study of 84 patients undergoing radiation segmentectomy reported a median treatment volume of 7.8% of total liver volume [29]. Even with ultra-selective radiation segmentectomy (i.e., targeting < 1 Couinaud segment), a retrospective study of 38 HCC patients found a median treatment volume of 4.5% of total liver volume [30]. These relatively larger treatment volumes may be associated with measurable regional liver function impairment and support a more prominent role for HBS in TARE compared with ablation, particularly for patient selection and dose planning. For example, van der Velden et al. demonstrated significant reductions in regional liver function within treated segments after yttrium-90 radioembolization, as measured by pre- and post-treatment HBS (median reduction: 2.1%/min/m^2^), while functional changes in non-treated segments were heterogeneous and not reliably predicted by volumetric assessment alone [31].

For stereotactic body radiotherapy (SBRT), a comparable rationale has been explored. In a pilot study, HBS revealed dose-dependent regional reductions in liver function after SBRT, enabling assessment of short-term functional liver toxicity beyond anatomical dose–volume metrics [32]. However, similar to ablation, research on the role of HBS in SBRT remains largely unexplored, precluding firm conclusions regarding routine clinical implementation.

HBS provides key advantages over CT- or MRI-based volumetry. Whereas volumetry quantifies anatomical volume and assumes uniform hepatic function, HBS provides a quantitative and spatially resolved measure of hepatocyte function [33]. This distinction is particularly important in cirrhotic or heterogeneously diseased livers, where volume often poorly reflects functional reserve, and in patients who have undergone prior liver-directed therapies that lead to uneven parenchymal function. In this study, there was no strong correlation between ablated non-tumor liver volume and absolute ablated liver function (Figure 3B). However, owing to the limited sample size, no formal statistical correlations between HBS and volumetric liver assessments could be performed, and the present findings should therefore be regarded as exploratory. This finding aligns with prior evidence demonstrating HBS superiority over volumetric assessment; in a retrospective study of 55 patients undergoing major liver resection (≥3 segments), HBS outperformed CT volumetry for predicting post-hepatectomy liver failure, demonstrating higher sensitivity (89% vs. 78%) and specificity (87% vs. 80%) [8].

While HBS provides valuable functional information, it also has limitations. Physiological factors can affect mebrofenin kinetics: because mebrofenin competes with bilirubin for hepatocyte uptake, hyperbilirubinemia may lead to underestimation of hepatic function. Likewise, low serum albumin, as plasma carrier of mebrofenin, can result in underestimated functional values.

This study has several important limitations. Its retrospective design introduces inherent limitations, including selection bias and unmeasured confounding. The small sample size, and heterogeneity in disease stage and prior treatments preclude definitive conclusions regarding the safety and efficacy of thermal ablation in patients with impaired liver function. HBS was performed for other clinical indications rather than as part of a standardized pre-ablation protocol, resulting in variable time intervals between HBS and ablation (median 1.0 months) and further contributing to potential selection bias. Potential shrinkage of the ablated tissue was not taken into account, which may have led to an underestimation of the amount of ablated healthy liver tissue [34,35]. Furthermore, quantification of absolute ablated liver function assumed a homogeneous distribution of liver function across the parenchyma. While this assumption decreases accuracy, the small ablation volumes involved (median 0.9% of total liver volume) are expected to minimize the impact of functional heterogeneity. Finally, despite establishing pre-defined criteria for assessing ablation-related hepatic decompensation, distinguishing decompensation directly attributable to thermal ablation from that caused by underlying disease progression remains inherently challenging.

Nonetheless, this pilot study was designed as a novel, exploratory investigation to explore and describe the potential role of HBS in guiding patient selection and risk stratification for thermal ablation in HCC, an area that has not previously been investigated, rather than to advocate its routine clinical use. Given the absence of strong correlations between HBS-derived liver function and either ALBI score or ablated non-tumor volume (Figure 3), HBS may provide complementary information beyond standard liver function laboratory assessment and baseline CT or MRI. Among patients who developed hepatic decompensation (all Child–Pugh class B), the likelihood of ablation-related decompensation was low given the time interval > 3 months between ablation and decompensation and the small ablation volumes. Therefore, the clinical value of HBS in thermal ablation may be limited to specific cases, particularly in patients at risk for compromised liver function (e.g., Child–Pugh B or greater, elevated ALBI scores, or borderline functional reserve following prior liver-directed therapy or surgery) in whom large-volume ablations are anticipated. However, given the limited sample size and exploratory nature of this pilot study, formal indications for the use of HBS in the setting of thermal ablation cannot be defined. To more accurately assess the added value of HBS in such high-risk patients, a prospective study with standardized pre- and post-procedural HBS is warranted to quantify global and regional changes in hepatic function and correlate these findings with clinical outcomes. Overall, our findings suggest that thermal ablation is safe even in patients with impaired liver function, and routine use of pre-ablation HBS does not appear necessary for standard thermal ablation practice.

## 5. Conclusions

HBS for thermal ablation is an unexplored area. In this pilot study, thermal ablation of HCC performed using the HepACAGA technique led to a median loss of 0.9% of functional liver parenchyma and appeared feasible and safe even in patients with severely impaired hepatic function on HBS. Routine pre-ablation HBS is therefore not recommended for standard ablation practice, but it may provide added value in high-risk patients undergoing large-volume ablations. This warrants further investigation.

## Figures and Tables

**Table 1:** Patient demographics and baseline characteristics.

Pt #	Sex (M/F)	Age (Years)	ECOG Score	BCLC Score	Cirrhosis (Etiology)	Child–Pugh Score	Total Bilirubin (µmol/L)	INR	ALBI Grade	MELD Score	Months Between HBS and Ablation	Liver Function on HBS [%/min/m^2^]
1	M	70	1	A	Yes, MASH	CP B7	34	1.2	2	-	16.3 Mo	1.6
2	M	61	0	A	Yes, post-HCV	CP B7	39	1.4	2	24	1.0 Mo	1.9
3	F	73	1	B	Yes, post-alcoholic	CP B7	16	1.1	2	8	10.1 Mo	2.5
4	M	81	-	0	Yes, post-alcoholic	CP B9	42	1.3	2	11	0.2 Mo	2.9
5	M	84	1	A	Yes, MASH	CP B8	8	1.3	1	13	2.1 Mo	3.2
6	M	65	1	B	Yes, MASH	CP B7	13	1.0	1	7	1.2 Mo	5.5
7	M	75	2	B	Yes, Sarcoidosis	CP A5	19	1.0	3	11	0.4 Mo	5.9
8	M	74	3	A	Yes, MASH	CP A5	7	1.3	1	10	0.8 Mo	6.4
9	M	78	1	A	Yes, post-HBV	CP A6	9	1.1	1	7	0.2 Mo	6.8

BCLC: Barcelona-Clinic Liver Cancer; CP: Child–Pugh; ECOG: Eastern Cooperative Oncology Group; F: Female; HBV: Hepatitis B virus; HBS: Hepatobiliary scintigraphy; HCV: Hepatitis C virus; INR = International Normalized Ratio; M: Male; MASH: Metabolic dysfunction-associated steatohepatitis; MELD: Model for End-Stage Liver Disease; Mo: Months; Pt #: Patient number.

**Table 2:** Procedural characteristics and volumetric analysis.

Pt #	# of Ablated Tumors	Tumor Size [mm]	$V_{\mathrm{total}\;\mathrm{liver}}$[cm^3^]	$V_{\mathrm{total}\;\mathrm{tumor}}$[cm^3^]	$V_{\mathrm{total}\;\mathrm{ablation}\;\mathrm{zone}}$[cm^3^] $(\%V_{\mathrm{ablated}\;\mathrm{liver}}$)	$V_{\mathrm{ablated}\;non\text{-}tumor}$* [cm^3^] $(\%V_{\mathrm{ablated}\;non\text{-}tumor}$)	Absolute Ablated Function ^†^ [%/min/m^2^] (% of Total Liver Function)
1	1	40	1560	21.0	65.8 (4.2%)	44.8 (2.9%)	0.046 (2.9%)
2	2	22, 9	766	6.0	34.0 (4.4%)	28.0 (3.7%)	0.071 (3.7%)
3	1	24	1450	7.2	20.5 (1.4%)	13.3 (0.9%)	0.023 (0.9%)
4	2	17, 11	1493	2.5	25.5 (1.7%)	23.0 (1.5%)	0.044 (1.5%)
5	1	12	1358	1.2	17.2 (1.3%)	16.0 (1.2%)	0.038 (1.2%)
6	3	27, 23, 16	2009	17.4	61.4 (3.1%)	44.0 (2.2%)	0.121 (2.2%)
7	1	47	1299	30.4	77.1 (5.9%)	46.7 (3.6%)	0.212 (3.6%)
8	1	22	1693	5.6	18.3 (1.1%)	12.7 (0.8%)	0.051 (0.8%)
9	1	25	1415	6.1	42.7 (3.0%)	36.6 (2.6%)	0.177 (2.6%)

Pt #: Patient number. * The volume of ablated non-tumor liver tissue ($V_{\mathrm{ablated}\;non\text{-}tumor}$) was calculated by subtracting the total tumor volume ($V_{\mathrm{total}\;\mathrm{tumor}}$) from the total ablation zone volume ($V_{\mathrm{total}\;\mathrm{ablation}\;\mathrm{zone}}$). ^†^ Absolute ablated function represents the proportion of total liver function (as measured by HBS) that was ablated, assuming homogeneous anatomical distribution of liver function across the parenchyma.

## Data Availability

The data presented in this study are available upon request from the corresponding author.
